# An Audit of Practice Quality and Alignment With Evidence Based Standards

**DOI:** 10.1192/bjo.2026.11725

**Published:** 2026-06-30

**Authors:** Mohamed Elsout, Ahmed Ahmed, Katey Beggan, Rebecca Evans, Snigdha Kota

**Affiliations:** 1 Cwm Taff Morgannwg University Health Board, Rhondda Cynon Taf, United Kingdom; 2 Swansea Bay University Health Board, Swansea, United Kingdom; 3 Cardiff and Vale University Health Board, Cardiff, United Kingdom

## Abstract

**Aims:**

**Additional Author Dr Bethany Ranjit Consultant Psychiatrist.**

To evaluate compliance with NICE national standards for physical health monitoring in adults receiving ADHD medication in the community setting along with reviewing the side effects profiles and follow up intervals.

NHS England commissioned a project to consider ADHD service provision.This highlighted a significant number of challenges our ADHD services face with increases in demand for ADHD services. In tandem with this NICE recommends that physical health monitoring (blood pressure, heart rate and weight) should take place prior to initiating medication and at a minimum of six-monthly intervals, alongside structured side-effect reviews. Given the significant increases in the outpatient ADHD patient caseloads, we were concerned about the documentation and monitoring requirements of this demographic of patients.

**Methods:**

A retrospective cohort audit of **207** adults prescribed ADHD medication by YCC CMHT was undertaken. Data extracted included demographic details, medication type and dose, dates and completeness of physical observation checks, and physical comorbidities. Additional variables collected included duration on stable dose, carecoordination status and psychiatric comorbidities. These were included to assess suitability for sharedcare transition.

**Results:**

The sample included **105** males and **102** females, with a mean age of **36.1** years. Medications prescribed were **Lisdexamfetamine** **63.3**%, methylphenidate XL 20.3%, methylphenidate IR **4.3%**, guanfacine **3.9**%, **Atomoxetine** **2.9**% and **Dexamphetamine** **0.5**%. **60%** of patients had been on a stable dose for more than six months. Physical health observations were recorded for **174 patients 84.1%**, although documentation was frequently incomplete, with missing pulse or weight entries. No standardised monitoring tool was used. Monitoring intervals were within NICE recommendations for **30.9%**, whereas **28.5%** were reviewed between 6–12 months, **24.6%** beyond 12 months and **15.9%** had no monitoring recorded. Psychiatric comorbidities were present in **37%** of patients. Physical comorbidities included Asthma **7.2%**, Hypertension **2.8%**, Fibromyalgia **2.4%**, Polycystic ovarian syndrome **1.9%** and Hypothyroidism **1.9%**.

**Conclusion:**

A substantial proportion of patients did not receive physical health monitoring within recommended intervals, and documentation quality was inconsistent and varied in detail. The absence of standardised tools likely contributed to underreporting of relevant adverse effects. Operational challenges, including limited access to monitoring equipment and continued prescribing despite nonattendance, were notable barriers. Implementing electronic prompts and adopting standardised monitoring templates may improve adherence to guidelines and enhance patient safety. Whilst also enhancing patient suitability for shared-care transitions.

